# Is abeta a sufficient biomarker for monitoring anti-abeta clinical studies? A critical review

**DOI:** 10.3389/fnagi.2013.00025

**Published:** 2013-07-02

**Authors:** Jens Moreth, Chrystelle Mavoungou, Katharina Schindowski

**Affiliations:** Faculty for Biotechnology, Institute of Applied Biotechnology, Biberach University of Applied ScienceBiberach/Riss, Germany

**Keywords:** passive immunization, dementia, therapeutic monoclonal antibodies, regulatory strategy, CSF, plasma increase, mode of action, pharmacogenetics and pharmacogenomics

## Abstract

Amyloid-beta (Aβ) in Alzheimer's disease (AD) appeared to be a promising target for disease-modifying therapeutic strategies like passive immunotherapy with anti-Aβ monoclonal antibodies (mAbs). Biochemical markers in cerebrospinal fluid (CSF) include alterations of Aβ that allow the diagnosis of AD. Biomarker strategies, such as the levels of Aβ in CSF and plasma, currently play an important role in early clinical trials for AD. Indeed, these strategies have a relevant impact on the outcome of such studies, since the biomarkers are used to monitor the bioactivity of anti-Aβ mAbs. The clinical trials of Solanezumab were mainly based on the readout of Aβ levels in CSF and plasma, whereas those of Bapineuzumab were based on cognition; however, little is known about the mechanisms altering these biomarker levels, and no biomarker has yet been proven to be a successful predictor for AD therapy. In addition, the Aβ biomarkers allow for the determination of free and bound anti-Aβ mAb in order to monitor the available amount of bioactive drug and could give hints to the mechanism of action. In this review, we discuss clinical Aβ biomarker data and the latest regulatory strategies.

## Aβ-aggregates and their implications on immunization

With about 70% of all cases, Alzheimer's disease (AD) is the most-common form of dementia (Alzheimer's Disease International, 2009) and countries in demographic transition will experience the greatest growth. AD is defined as a multifactorial disease with the pathogenic cerebral deposition of the aggregated proteins Amyloid-β (Aβ) and hyper-phosphorylated tau (phospho-tau). Despite the well-accepted pathogenic role of Aβ (Selkoe, 2001), the underlying pathogenic mechanism is still elusive (Broersen et al., 2010). Aβ-aggregates—majorly derived from Aβ_40_ and Aβ_42_—are generated from amyloid precursor protein by sequential proteolysis (Haass and Selkoe, 2007) followed by self-association from monomeric to soluble oligomeric and protofibrillar Aβ. Protofibrillar Aβ further aggregates into insoluble Aβ-fibrils and deposits in the brain as amyloid plaques. Since the number of these plaques does not correlate well with the severity of dementia (Terry, 2006)—as opposed to soluble Aβ-aggregates (McDonald et al., 2010)—the amyloid hypothesis has been reformulated, positioning soluble Aβ aggregates as hallmark in AD pathology (Walsh and Selkoe, 2007; Broersen et al., 2010). A plethora of different Aβ-species with overlapping size and morphology have been described (Broersen et al., 2010; Benilova et al., 2012): Aβ-dimers (Shankar et al., 2008; O'Nuallain et al., 2010), low-molecular weight oligomers comprising dimeric to tetrameric Aβ (Walsh et al., 2005), pentamers and hexamers (Ahmed et al., 2010), dodecameric Aβ56^*^ (Lesne et al., 2006; Reed et al., 2011), globulomers (Barghorn et al., 2005), Aβ-oligomers (Kayed et al., 2003), Alzheimer-derived diffusible ligands (ADDLs; Lambert et al., 1998), protofibrils (Walsh et al., 1999), and amylospheroids (Hoshi et al., 2003). Although, the size and molecular weight of these Aβ-species have predominantly been used for differentiation, the peptide source, either synthetic or endogenous, and the applied methods for characterization—e.g., SDS-PAGE, TEM, AFM, Ultracentrifugation—hamper a direct comparison (Moreth et al., 2013). Despite the pathological relevance of endogenous Aβ-species, low protein concentrations and protein heterogeneity elude a precise characterization of the molecular identity. The synthetic Aβ-aggregate is applicable to a more-precise characterization, but still retains limited relevance, since the variety of reported Aβ-aggregates has yet to be proven to be present in AD brain. Furthermore, the identification of Aβ-aggregates is hampered owing to their meta-stability and the ability for inter-conversion in different aggregation pathways (Moreth et al., 2013), which was also mentioned by Bitan et al. (2005). This is of great importance for immunization, since the fate of the pre-aggregated Aβ is elusive after injection.

## Occurrence of Aβ species in plasma and CSF

From a set of upcoming biomarkers (Fagan and Perrin, 2012), the most-established biomarkers for AD diagnosis in cerebrospinal fluid (CSF) are still the determination of Aβ_42_, total-Tau and phospho-Tau_181_ (Di Carlo et al., 2012). Only a combination of these three CSF biomarkers increases the validity of the diagnosis with a combined sensitivity of 95% (Blennow et al., 2010). In AD, CSF-Aβ_42_ is significantly decreased, which is believed to be due to decreased clearance of aggregated Aβ_42_ from the brain. The Aβ_40_ levels seem to be constant and therefore the increased Aβ_42_/Aβ_40_ ratio has been suggested to improve early AD-diagnosis. However, this is still controversial and for plasma-derived Aβ reports are even more contradictory (Zetterberg, 2008; Zetterberg et al., 2010). To mention the prefibrillar Aβ-aggregates as the prime toxic agents in AD, one might address these as potential biomarkers. However, there is still a lack of a robust method for the detection of larger Aβ-aggregates *in vivo* (e.g., ADDL, Aβ-oligomers). Some recent reports showed methods for Aβ-aggregate detection based on ELISA, IP western blotting and Aβ-aggregate capture assays. All of these methods are based on conformation-specific antibodies, which do not detect monomeric or fibrillar, but rather the prefibrillar aggregates (Funke et al., 2009), even though the most relevant Aβ-aggregate for AD diagnosis is still elusive. Furthermore, based on the described meta-stability of Aβ-aggregates (Moreth et al., 2013), it might be misleading to focus on a single aggregate species if the whole spectrum of aggregates from the dimer up to protofibrillar Aβ are present in the brain and of importance in AD-progression.

## Plasma and CSF Aβ as biomarkers to monitor passive anti-Aβ immunotherapy clinical studies

Aβ has a complex pharmacokinetic profile, as it is permanently produced in brain as well as in the periphery, and transported back and forth between both pharmacokinetic compartments (Zlokovic et al., 1993; Ghersi-Egea et al., 1996; Shibata et al., 2000). Soluble Aβ is either degraded by proteases (Iwata et al., 2005), transported via the blood-brain barrier by receptors like LRP (Sagare et al., 2007), RAGE (Deane et al., 2003), and P-glycoprotein (Ito et al., 2006), or aggregates to multimers and plaques. Likewise, plaque Aβ is in steady-state equilibrium with soluble Aβ (Kawarabayashi et al., 2001). Finally, Aβ is rapidly eliminated by hepatic and renal degradation (Ghiso et al., 2004). PET scanning with the Pittsburgh compound (PiB) detects fibrillar Aβ. CSF Aβ_42_ and PET measures of fibrillar Aβ are significantly inversely correlated with each other, likely to reflect Aβ deposition in the brain (Fagan et al., 2006).

**Figure 1:**
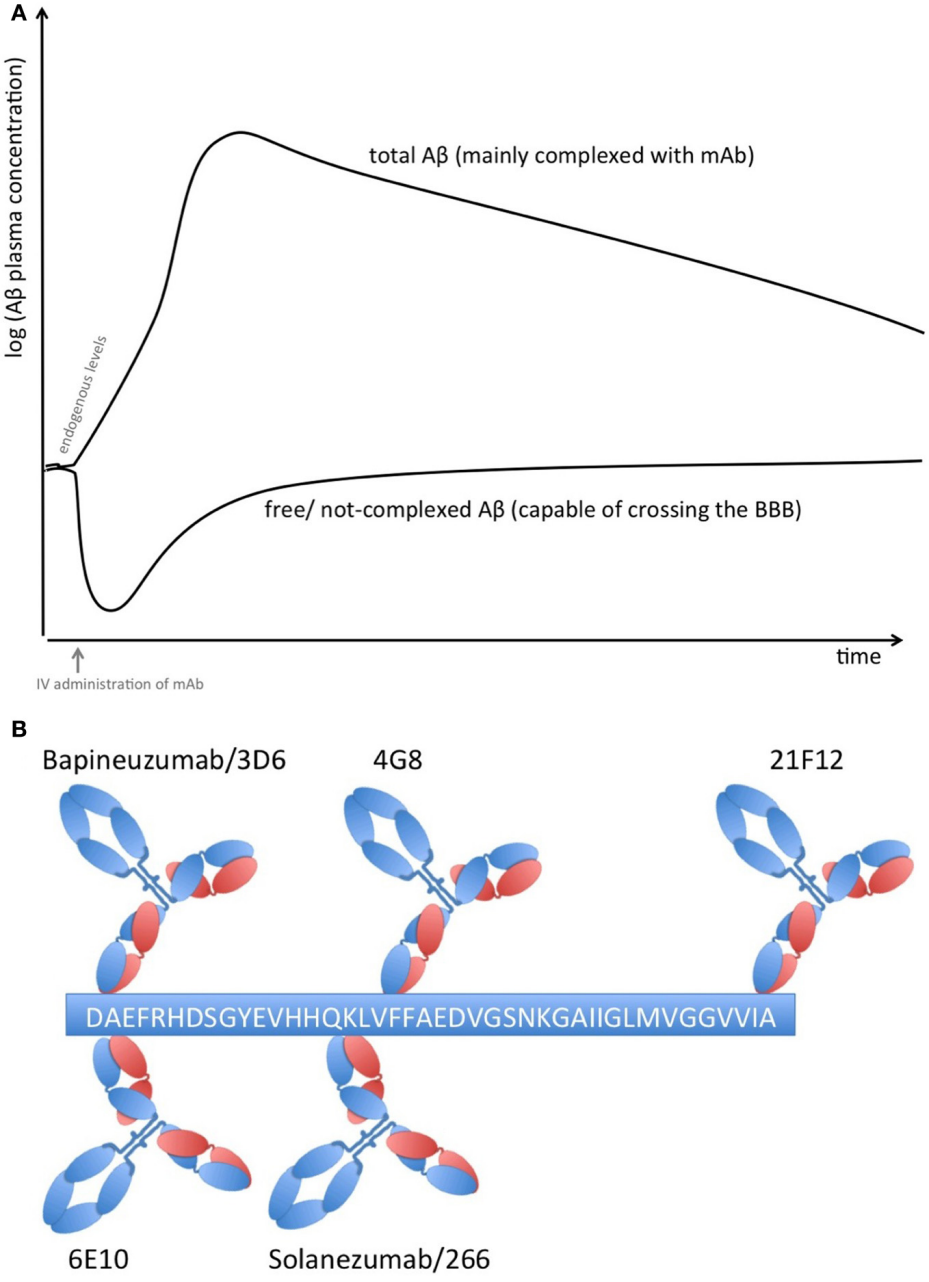
**(A)** Plasma Aβ levels after treatment with an Aβ sequestering compound. Anti-Aβ mAbs capture soluble Aβ and form Aβ-mAb complexes, which have a much longer half-life than free Aβ alone. Therefore, total Aβ (i.e., free and bound) plasma levels rise while free Aβ levels drop rapidly but return rather quickly to normal levels due to its rapid synthesis in many tissues. **(B)** Binding sites on Aβ_1−42_ of therapeutic and diagnostic mAbs. Adapted from Johnson-Wood et al. (1997); Clarke and Shearman (2000).

Proteins in plasma, like antibodies that capture soluble Aβ, are capable of sequestering soluble forms of Aβ from their bound and circulating forms. Total Aβ plasma levels will therefore increase while free Aβ levels reduce due to the longer half-life of protein-complexed Aβ [see Figure 1A; (Park et al., 2012)]. The elimination of the Aβ-protein complex is according to the complex's half-life, which is rather long in the case of FcRn-recycled monoclonal antibodies (mAbs). Complexed Aβ is predictably not transported across the blood brain barrier, does not form multimers, and influences the equilibrium between soluble Aβ and plaque Aβ that appears to result in improved clearance of cerebral Aβ, e.g., CSF Aβ. The Aβ-binding proteins should have an affinity to Aβ high enough to compete with endogenous Aβ-binding proteins and transporters. Free Aβ drops rapidly after Aβ is sequestered, but due to its rapid synthesis in various tissues, it is restored to basal endogenous levels rather quickly (Barten et al., 2005)

Peripherally-administered mAbs that sequester soluble Aβ result in an increase of plasma Aβ (DeMattos et al., 2002) that is correlated to its affinity; some mAbs are even capable of reducing CSF Aβ (Mavoungou and Schindowski, 2013). Several studies used these biomarkers as clinical strategy (Table 1). Solanezumab caused a sharp, sustained, and dose-dependent increase of plasma Aβ_1−40_ and Aβ_1−42_ (Farlow et al., 2012). CSF Aβ_1−40_ and Aβ_1−42_ increased in the mild to moderate AD patients with 0.1% of plasma levels of Solanezumab found in the CSF. The rise in level of total Aβ in plasma and CSF is assumed to be related to target engagement (Strobel and Bowman Rogers, 2012). Free CSF Aβ was determined by protein G sepharose immunoprecipitation to deplete immunoglobins and subsequent ELISA (Farlow et al., 2012). Therefore, this method was used for CSF samples only, since immunoglobulin plasma concentrations are too high for this method. In a rather small cohort of patients, free CSF Aβ_1−40_ decreased with treatment, while free Aβ_1−42_ did not. It is suspected that the higher amount of free CSF Aβ_1−42_ is related to the dissolution of plaques that were mainly composed of Aβ_42_. However, PiB scans of another subcohort showed no significant change between the groups, although treated patients with mild AD had a trend toward less amyloid, this lacked statistical significance (Matthews and Bader, 2012).

**Table 1:** **Clinical effects of anti-Aβ mAbs on CSF and plasma Aβ, adapted from Mavoungou and Schindowski (2013)**.

**Study/cohort**	**Subcohort size for biomarker evaluation**	**Evaluated biomarker**	**Clinical effect of treatment on biomarker**	**Clinical effect on cognition**	**PK data of mAb**	**References**
**BAPINEUZUMAB (HUMANIZED 3D6)**						
201 Phase II	Placebo: *n* = 14	CSF Aß_*x*−42_	No changes	In small cohort 6% less loss of ADAS-Cog scores after 18 months	Approximately 0.3%	Salloway et al., 2009
	BAPI: *n* = 20	Total CSF tau	No changes			
		CSF phospho-tau	Trend to reduction (*p* = 0.056)		CSF-plasma ratio
Phase II: pooled 201 and 202	Placebo: *n* = 19	CSFAß_1−40_	No changes	Not determined	Not determined	Blennow et al., 2012
	BAPI: *n* = 26−27	CSF Aß_*x*−42_	Decrease from baseline			
		CSF Aß_1−42_	No changes			
		Total CSF tau	No changes			
		CSF phospho-tau	Reduction (*p* = 0.03)			
Phase III: 301 *(ApoE4* carrier)	Placebo: *n* = 77	CSF phospho-tau	No changes at 0.5 mg/kg	In a subcohort of mild cases at 1.0 mg/kg ~30% less loss of DAD scores after 18 months	Not determined	Salloway et al., 2012
	0.5mg/kg: *n* = 47	CSF phospho-tau	Reduction at 1.0 mg/kg			
	1.0mg/kg: *n* = 54					
Phase III: 302 *(ApoE4* non-carrier)	Placebo: *n* = 85	CSF phospho-tau	Reduction at 0.5 mg/kg	No effect on cognition after 18 months, even not for mild cases	Not determined	Sperling et al., 2012
	0.5mg/kg: *n* = 127					
**SOLANEZUMAB (HUMANIZED m266)**						
Phase II	Placebo: *n* = 8;	CSF total Aß_40_	Increase at high dose	No significant cognitive benefit on the ADAS-cog score over after 12-weeks	0.1%	Farlow et al., 2012
	SOLA: *n* = 10−11 per dose group	CSF total Aß_42_	Increase at high dose		CSF-plasma ratio	
		CSF free Aß_40_	Decrease at high dose			
		CSF free Aß_42_	Increase at high dose			
		Plasma total Aß_40_	Dose-dependent increase			
		Plasma total Aß_42_	Dose-dependent decrease			
**GSK933776 (DISCONTINUED FOR AD)**						
Phase I	Placebo: *n* = 14;	Plasma total Aß	Dose-dependent increase	Not determined	>0.2%	GlaxoSmithKline, 2011
	GSK933776: *n* = 3−6 per dose group	Plasma free Aß	Dose-dependent decrease		CSF-plasma ratio	
		CSF Aß_1−38_ tau/phospho-tau	Increase at the highest dose			
			No changes			
**CRENEZUMAB (MABT5 102A)**						
Phase I	MABT: *n* = 25−31 per regime group	Plasma total Aß_40_	Dose-dependent increase	Not determined	Not determined	Adolfsson et al., 2012
		Plasma total Aß_40_	Dose-dependent increase			

The clinical biomarker data from Bapineuzumab are more difficult to interpret, due to the fact that Bapineuzumab binds both soluble and plaque Aβ, and the methodological strategy is rather unclear. Aβ_1−40_ and Aβ_x−42_ were detected by a sandwich ELISA using 4G8 for capture and a C-terminal mAb for detection (Figure 1B). 4G8 does not interfere with Bapineuzumab binding (Johnson-Wood et al., 1997; Clarke and Shearman, 2000). Interestingly, Aβ_1−42_ was determined with an ELISA using 3D6 as capture. 3D6 is the parental molecule of Bapineuzumab and therefore these two mAbs compete with each other when binding Aβ. Consequently, Bapineuzumab-Aβ complexes in CSF will predictably not be detected in this assay, though according to PK data Bapineuzumab occurs in CSF with 0.3% incidence of plasma levels (Blennow et al., 2012). Hence, the clinical data reveal no changes in CSF Aβ_1−42_ levels with Bapineuzumab treatment, while Solanezumab treatment revealed an increase in Aβ_1−42_ detected with the C-terminal mAb 21F12 and the N-terminal 3D6. Moreover, to avoid signal suppression due to steric hindrance, the authors of the Solanezumab study spiked an excess of Solanezumab in the assay buffer (Farlow et al., 2012). Furthermore, Bapineuzumab treatment decreased CSF phospho-tau (Salloway et al., 2012; Sperling et al., 2012). Like Solanezumab, Bapineuzumab was not active on patient's cognition and activities of daily living unless subsequent post-test of subcohorts were considered for re-analysis (Salloway et al., 2009; Lilly, 2012; Matthews and Bader, 2012). In summary, both antibodies engaged their target, but they hardly improved clinical signs (Strobel and Bowman Rogers, 2012). Bapineuzumab's clinical development was discontinued for AD in 2012 (Johnson & Johnson, 2012), AAB-003/PF-0523681 an engineered 3D6 replaced Bapineuzumab in the sponsor's pipeline (Pfizer, 2013). Dose-dependent plasma total Aβ increases were reported from GSK933776 and Crenezumab with decreased free plasma Aβ levels (GlaxoSmithKline, 2011; Adolfsson et al., 2012).

## The importance of an appropriate biomarker strategy for AD

In an ideal world with a successful anti-AD therapy, the detection of AD biomarkers should indicate appropriate patient selection likely to derive therapeutic benefit. The EMA tried first to get closer to this ideal world, at least from the regulatory side, and introduced research diagnostic criteria that added specificity to the prevailing concept of mild cognitive impairment (Dubois et al., 2007). This set the stage for new types of trials (Strobel and Bowman Rogers, 2012). The criteria are closer to the disease, combining a mild but measurable memory impairment with a biomarker change. The EMA considered firstly that a pathological signature based on CSF Aβ_42_ and phospho-tau was acceptable for identifying prodromal-stage patients who are at risk of developing AD (European Medicines Agency, 2011b), secondly, using hippocampal volume (European Medicines Agency, 2011a), and thirdly, using amyloid PET as a biomarker to enrich pre-dementia trials (European Medicines Agency, 2011c). Likewise, the FDA revised its criteria as well. Nevertheless, evidence is needed that a surrogate marker predicts a subsequent clinical outcome. Qualifying disease- and disorder-specific biomarkers for AD can still be considered “exploratory” from a regulatory point of view, therefore making an accurate validation and qualification questionable. Nevertheless, biomarkers, in particular those appropriate to guide selection of patients for clinical trials as well as those used as surrogate endpoint for drug efficacy, have reached the status of “probable valid biomarker” within the scope of investigation drugs along with an effective clinical trial strategy. In conclusion, the results show that CSF biomarkers are better predictors of progression to AD than plasma Aβ isoforms (Hansson et al., 2010)

Florbetapir, which binds Aβ plaques like PiB, was fast-track reviewed by the FDA and is currently the first granted and therefore qualified imaging agent for clinical use (Food and Drug Administration, 2012a). Following the results of the evaluation, even though a positive scan indicates moderate to frequent plaques, a positive Florbetapir scan is not AD specific, indicating that it is not appropriate to establish an accurate diagnosis of AD (Food and Drug Administration, 2012b). In fact, nobody currently knows why cognitively normal people accumulate Aβ in their brains, and what that might mean for their future brain health. The AD Neuroimaging Initiative (ADNI) belongs to one of the instruments to gain more information on the disease by means of PET and MRI linked to genetic disposition, cognitive impairment as well as CSF and plasma biomarkers. The use of such information obviously is crucial to set future clinical designs for AD (Food and Drug Administration, 2012a) but also as prophylactic examination for physicians in case of genetic predisposition for AD. On the other hand, exploring a set of imaging and biochemical biomarkers helps to develop regulatory guidelines to change diagnostic criteria, their validation and finally to support the potential use of biomarkers in different stages of drug development.

While the expressed view is that CSF biomarkers indicate the pathologic processes underlying AD, it is also important to keep in mind that specific genotypes like *ApoE4* and presenilin mutations affect the degree of pathological change. Therefore, using pharmacogenetics will enrich clinical drug development. From the presented data it seems that use of CSF markers is an unavoidable step for a correct and early diagnosis. However, the data reported show only the positive results, with no negative comments or discussion on potential pitfalls. Uncritical support without showing areas of uncertainty or controversy could be misleading, in helping to improve the design of subsequent randomized controlled clinical trials. The hazard ratio in longitudinal studies shows an extremely large confidence interval, which is not that supportive for the utility of monitoring. The specifications of the confidence interval for such a multifactorial disease like AD might be nowadays too tight in the light of the recent findings about the disease. That means it is understandable that the confidence interval cannot be met for most of the cases. A combination of biomarkers to boost the sensitivity and reliability for tracking AD progression at different stage and widening the current specification limits with respect to confidential interval would better match with the variability of the results.

## Conclusion

To summarize, Aβ-aggregates reveal a remarkable metastability and the ability for reorganization within different aggregate equilibria. One might assume that the whole spectrum of prefibrillar Aβ-aggregates is of relevance in AD. Thus, targeting one specific species of Aβ with immunotherapy and using Aβ as preclinical and clinical biomarker is based on tentative, though countless data that apparently do not reflect the clinical reality. Therefore, the clinical biomarker data from the phase II and III studies of the most-advanced anti-Aβ mAbs are not appropriate to predict the cognitive outcome, even though the results show that CSF Aβ appears to be more relevant than plasma Aβ. This stresses the urgent need to understand the molecular basis of AD and to find adequate surrogate biomarkers. From a regulatory point of view, the approval of a highly-innovative active substance for the treatment for AD still remains a challenge. Although biomarker strategies have been taken more and more into account, the current study designs for AD superficially address the silent pathogenesis of the disease. The EMA and FDA are looking forward to qualifying new surrogate endpoints that encompass appropriate biomarker concepts in support of a robust biomarker strategy, which would enable the discovery of medicinal products that are active in interfering with AD pathogenesis.

## Author contributions

Jens Moreth supported with novel data on Aβ conformation and aggregation, Chrystelle Mavoungou supported with insight from regulatory affairs and Katharina Schindowski supported with insight on Aβ immunotherapy und neuroimmunology. All authors drafted the manuscript. All authors read and approved the final manuscript.

### Conflict of interest statement

The authors declare that the research was conducted in the absence of any commercial or financial relationships that could be construed as a potential conflict of interest.

## Abbreviations

aa: amino acid
Aβ: amyloid-beta
AβO: Aβ oligomers
AD: Alzheimer's disease
ADAS-Cog: Alzheimer's Disease Assessment Scale-cognitive subscale
ADDLs: Alzheimer derived diffusible ligands
ADNI: Alzheimer's disease neuroimaging initiative
AFM: atomic force microscopy
ApoE4: ApolipoproteinE4
CSF: cerebrospinal fluid
DAD: disability assessment for dementia
EMA: European Medicine Agency
FDA: food and drug administration
J&J: Johnson&Johnson
LRP: low density lipoprotein receptor-related protein
MRI: magnetic resonance imaging
PET: positron emission tomography
phospho-tau: hyperphosphorylated tau
PK: pharmacokinetic
SDS-PAGE: sodium dodecyl sulfate polyacrylamide gel electrophoresis
TEM: transmission electron microscopy.
